# Influential factors and spatial–temporal distribution of tuberculosis in mainland China

**DOI:** 10.1038/s41598-021-85781-7

**Published:** 2021-03-18

**Authors:** Siyu Bie, Xijian Hu, Huiguo Zhang, Kai Wang, Zhihui Dou

**Affiliations:** 1 Department of Mathematics and Systems Science, Xinjiang University, Urumqi, 830046 China; 2 Department of Medical Engineering and Technology, Xinjiang Medical University, Urumqi, 830011 China; 3 Chinese Center for Disease Control and Prevention, Beijing, 102206 China

**Keywords:** Tuberculosis, Statistics

## Abstract

Tuberculosis (TB) is an infectious disease that threatens human safety. Mainland China is an area with a high incidence of tuberculosis, and the task of tuberculosis prevention and treatment is arduous. This paper aims to study the impact of seven influencing factors and spatial–temporal distribution of the relative risk (RR) of tuberculosis in mainland China using the spatial–temporal distribution model and INLA algorithm. The relative risks and confidence intervals (CI) corresponding to average relative humidity, monthly average precipitation, monthly average sunshine duration and monthly per capita GDP were 1.018 (95% CI 1.001–1.034), 1.014 (95% CI 1.006–1.023), 1.026 (95% CI 1.014–1.039) and 1.025 (95% CI 1.011–1.040). The relative risk for average temperature and pressure were 0.956 (95% CI 0.942–0.969) and 0.767 (95% CI 0.664–0.875). Spatially, the two provinces with the highest relative risks are Xinjiang and Guizhou, and the remaining provinces with higher relative risks were mostly concentrated in the Northwest and South China regions. Temporally, the relative risk decreased year by year from 2013 to 2015. It was higher from February to May each year and was most significant in March. It decreased from June to December. Average relative humidity, monthly average precipitation, monthly average sunshine duration and monthly per capita GDP had positive effects on the relative risk of tuberculosis. The average temperature and pressure had negative effects. The average wind speed had no significant effect. Mainland China should adapt measures to local conditions and develop tuberculosis prevention and control strategies based on the characteristics of different regions and time.

## Introduction

Tuberculosis ia a public health that places a serious burden on people in countries and regions around the world. According to the “Global Tuberculosis Report” in 2020^1^, 10 million people worldwide were infected with tuberculosis in 2019, and China had 840,000 people, ranking third. Although China has done a lot of work on the prevention and control of tuberculosis^2^, there are still many provinces^3–5^ in areas with a high incidence of tuberculosis. It is still necessary to strengthen the prevention and control of the occurrence and spread of tuberculosis.

The factors affecting tuberculosis are diverse^6^, including meteorological factors^7,8^, socioeconomic factors^9–12^ and so on. The incidence of tuberculosis has a significant seasonal feature^13,14^ and is closely related to geographic location^6^. Studies have analyzed spatial–temporal distribution characteristics of tuberculosis in mainland China^8,15–17^ and some provinces^18–21^ and other descriptive statistics^22^, but there are few monthly studies.

This paper studies the influencing factors and spatial–temporal distribution characteristics of tuberculosis in mainland China on the basis of existing studies^7,8^. Meteorological factors and socio-economic factors such as temperature, relative humidity, precipitation, sunshine duration, wind speed, air pressure, and monthly per capita gross domestic product (GDP) are selected to establish a Bayesian spatial–temporal distribution model and use the INLA algorithm to solve it. By analyzing the regression coefficients of influencing factors and relative risks in different time and locations, the influencing factors and spatial–temporal distribution characteristics of tuberculosis are explored, which provides theoretical basis for applying the INLA algorithm to spatial epidemiology and formulating scientific tuberculosis prevention and control measures in the future.

## Results

The five provinces with the highest cumulative incidence rate (100,000) in December 2013 were Xinjiang (170.349), Tibet (136.346), Guizhou (133.589), Qinghai (104.76) and Guangxi (96.217). The five highest provinces in 2014 were: Xinjiang (173.416), Tibet (145.220), Guizhou (129.173), Qinghai (100.257) and Guangxi (99.910). The five highest provinces in 2015 were: Xinjiang (179.716), Tibet (137.407), Guizhou (132.626), Qinghai (122.310) and Hainan (97.113). This article studies the relative risk of tuberculosis in mainland China. The results of the study are divided into five aspects: spatial stratified heterogeneity detection, meteorological and social factors that affect the risk of tuberculosis, spatial distribution, temporal distribution and spatial–temporal distribution of relative risk of tuberculosis.

### Spatial stratified heterogeneity detection

The factors include: location, time, temperature, relative humidity, precipitation, duration of sunshine, wind speed, air pressure and per capita GDP. Detecting the spatial stratified heterogeneity of the incidence and influencing factors of tuberculosis from 2013 to 2015, the significance level is less than 0.1, and the q-statistics of factors are 0.875, 0.061, 0.006, 0.058, 0.021, 0.033, 0.029, 0.218 and 0.201, respectively. This shows that the factors studied in this article are all significant in explaining the distribution of tuberculosis, and the spatial effects has the strongest explanatory power. The *q*-statistics and *p*-value are shown in Table 1.

**Table 1:** Spatial stratified heterogeneity with q-statistic.

Factor	*q*-statistic	*p*-value	Factor	*q*-statistic	*p*-value
Location	0.875	0	Sunshine duration	0.033	0
Time	0.061	0	Wind speed	0.029	0
Temperature	0.006	0.085	Air pressure	0.218	0
Relative humidity	0.058	0	Per capita GDP	0.201	0
Precipitation	0.021	0	Location $\bigcap$ time	1	

### Meteorological and socioeconomic factors

The posterior results of the regression coefficients of meteorological and social factors are shown in Table 2. The relative risk of each factor is $RR_{factor}=exp(\beta )$. The posterior means of the regression coefficients of average relative humidity, monthly average precipitation, monthly average sunshine duration, and monthly per capita GDP are 0.018, 0.014, 0.026, and 0.025. The corresponding relative risks are 1.018 (95% CI 1.001–1.034), 1.014 (95% CI 1.006–1.023), 1.026 (95% CI 1.014–1.039), and 1.025 (95% CI 1.011–1.040). These four variables have significant positive effects on the incidence of tuberculosis. When the variable increases by one unit, the relative risks increase by 1.8% (95% CI 0.1–3.4%), 1.4% (95% CI 0.6–2.3%), 2.6% (95% CI 1.4–3.9%) and 2.5% (95% CI 1.1–4.0%).

**Table 2:** Posterior results.

Variable	Posterior mean (CI)	Standard deviation	Relative risk (CI)
Intercept	$-$ 0.063 ($-$ 0.077, $-$ 0.048)	0.007	0.939 (0.926, 0.953)
Average temperature	$-$ 0.045 ($-$ 0.059, $-$ 0.031)	0.007	0.956 (0.942, 0.969)
Average relative humidity	0.018 (0.001, 0.034)	0.008	1.018 (1.001, 1.034)
Monthly average precipitation	0.014 (0.005, 0.023)	0.005	1.014 (1.006, 1.023)
Monthly average sunshine duration	0.026 (0.014, 0.038)	0.006	1.026(1.014, 1.039)
Average wind speed	$-$ 0.009 ($-$ 0.020, 0.002)	0.006	0.991 (0.980, 1.002)
Average air pressure	$-$ 0.268 ($-$ 0.411, $-$ 0.133)	0.071	0.767 (0.664, 0.875)
Monthly per capita GDP	0.025 (0.011, 0.040)	0.007	1.025 (1.011, 1.040)

The posterior means of the regression coefficients of mean temperature and mean air pressure are − 0.045 and − 0.268. The corresponding relative risks are 0.956 (95% CI 0.942–0.969) and 0.767 (95% CI 0.664–0.875). The two have significant negative effects on the incidence of tuberculosis. When the variable increases by one unit, the relative risks reduce by 4.4% (95% CI 3.1–5.8%) and 23.3% (95% CI 12.5–33.6%).

The posterior mean of the regression coefficient of average wind speed is − 0.009, and the relative risk is 0.991 (95% CI 0.980–1.002). The 95% confidence interval for relative risk contains 1, so average wind speed has no significant effect on the incidence of tuberculosis. Note that the CI is the one under the assumption of the model, rather than the real error, if the assumption of the model is different from the property of a population.

### Spatial and temporal distribution

#### Spatial distribution

**Figure 1:**
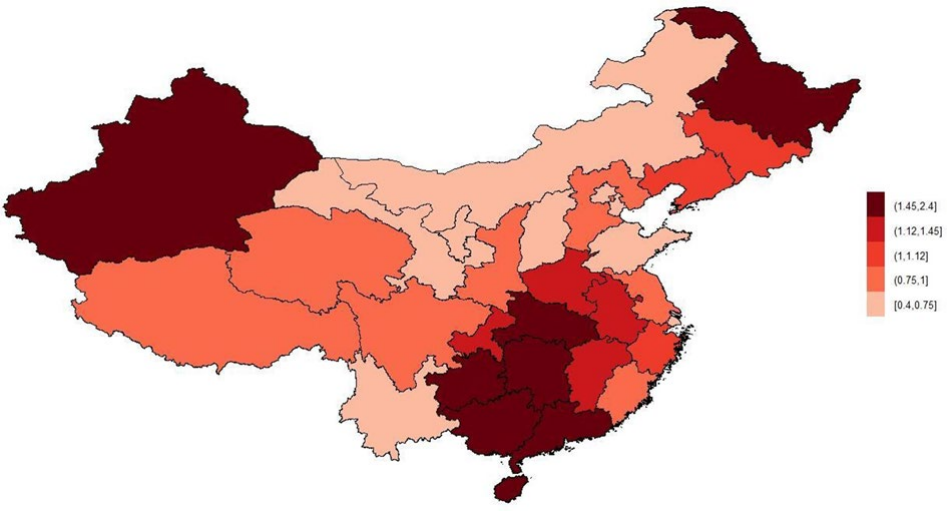
$$RR_{spatial}$$ distribution.

The relative risk in area is $RR_{spatial}$ = $exp(u+v)$. The relative risk $RR_{spatial}$ of spatial effects in 31 provinces is shown in Table 3 and Fig. 1. It can be seen from Fig. 1 that the regions with relatively high relative risks are the Northwest and South China regions, which means that the risk of tuberculosis is higher in these two regions. The five provinces with relatively high relative risk are: Xinjiang Uygur Autonomous Region, Guizhou Province, Hainan Province, Guangxi Zhuang Autonomous Region, and Hunan Province. The corresponding relative risks are 2.360 (95% CI 2.134–2.592), 2.028 (95% CI 1.921–2.135), 1.909 (95% CI 1.715–2.127), 1.886 (95% CI 1.733–2.058), and 1.657 (95% CI 1.518–1.814). The five provinces with relatively low spatial risk are: Beijing, Ningxia Hui Autonomous Region, Shandong Province, Shanghai, and Tianjin. These provinces are mostly in East and Central China, which means that these two regions have a lower risk of tuberculosis. On the whole, the relative risk of tuberculosis has obvious spatial differences, showing a trend of distribution in the south and light in the north. In the future, attention should be paid to the spread of tuberculosis in Xinjiang, Guangxi, Hainan, and Heilongjiang, as well as epidemic monitoring in high-risk areas such as Jiangxi, Chongqing, Henan and Anhui.

**Table 3:** $RR_{spatial}$ in 31 provinces.

Province	Anhui	Beijing	Chongqing	Fujian
RR (95%CI)	1.209 (1.090, 1.344)	0.612 (0.563, 0.666)	1.371 (1.305, 1.441)	0.854 (0.790, 0.924)
Province	Gansu	Guangdong	Guangxi	Guizhou
RR (95%CI)	0.745 (0.626, 0.871)	1.470 (1.319, 1.643)	1.886 (1.733, 2.058)	2.028 (1.921, 2.135)
Province	Hainan	Hebei	Heilongjiang	Henan
RR (95%CI)	1.909 (1.715, 2.127)	0.850 (0.807, 0.897)	1.591 (1.473, 1.722)	1.275 (1.172, 1.390)
Province	Hubei	Hunan	Jiangsu	Jiangxi
RR (95%CI)	1.532 (1.407, 1.672)	1.657 (1.518, 1.814)	0.837 (0.740, 0.947)	1.417 (1.278, 1.575)
Province	Jilin	Liaoning	Inner Mongolia	Ningxia
RR (95%CI)	1.057 (0.989, 1.133)	1.073 (0.975, 1.183)	0.748 (0.711, 0.785)	0.589 (0.521, 0.661)
Province	Qinghai	Shaanxi	Shandong	Shanghai
RR (95%CI)	0.839 (0.552, 1.198)	0.890 (0.853, 0.927)	0.684 (0.618, 0.760)	0.572 (0.504, 0.651)
Province	Shanxi	Sichuan	Tianjin	Xinjiang
RR (95%CI)	0.724 (0.694, 0.755)	0.794 (0.646, 0.957)	0.402 (0.355, 0.455)	2.360 (2.134, 2.592)
Province	Tibet	Yunnan	Zhejiang	
RR (95%CI)	0.991 (0.619, 1.474)	0.706 (0.616, 0.801)	1.032 (0.926, 1.153)	

#### Temporal distribution

**Figure 2:**
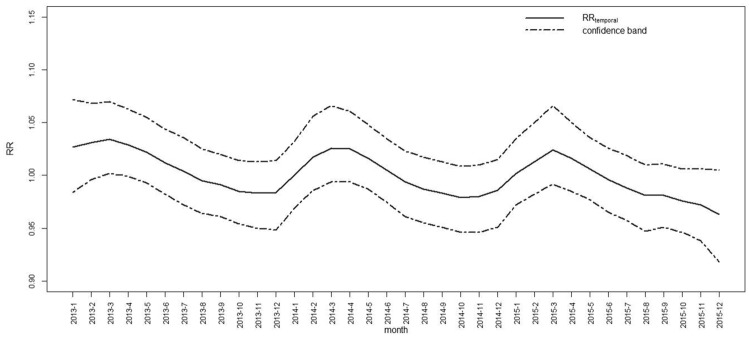
$$RR_{temporal}$$ for 36 months.

**Figure 3:**
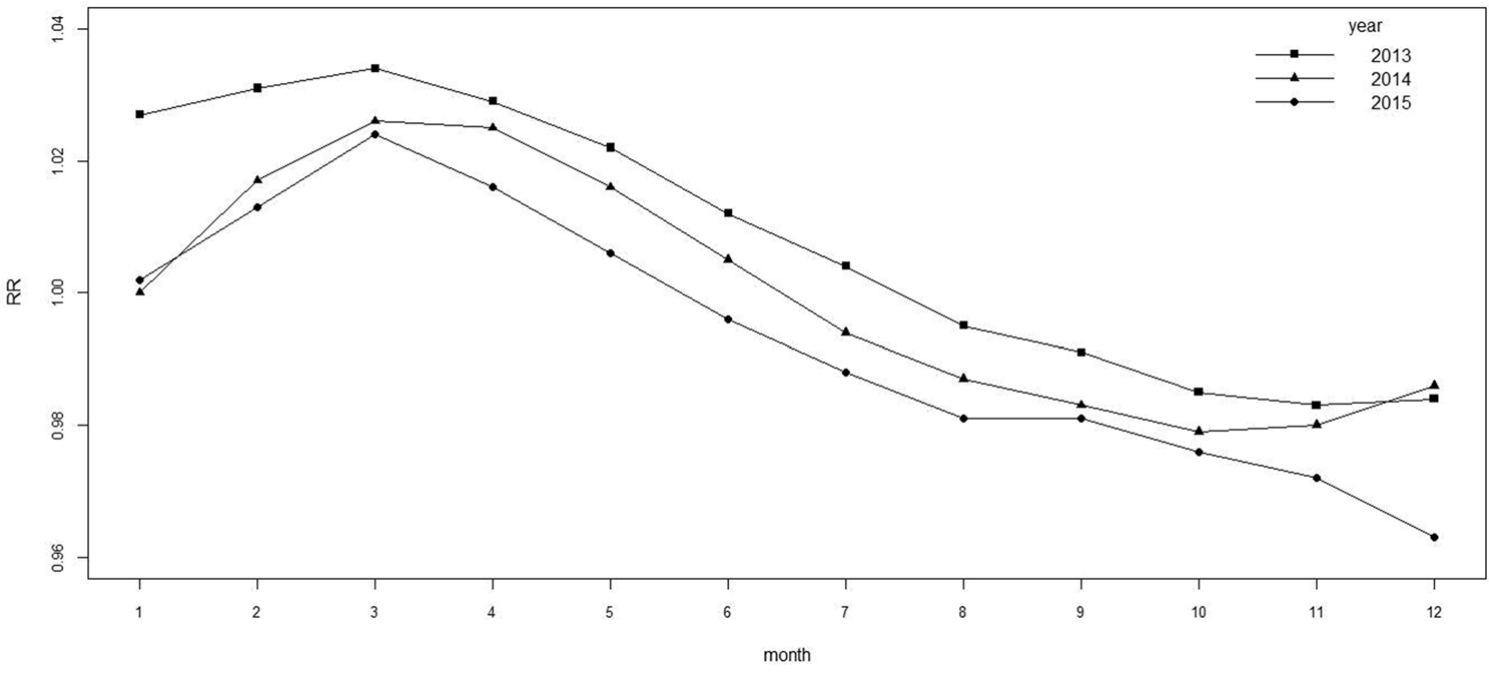
$RR_{temporal}$ for each year.

The relative risk in time is $RR_{temporal}$ = $exp(\gamma +\varphi )$. The relative risk $RR_{temporal}$ of temporal effects is shown in Figs. 2 and 3. Figure 2 shows the relative risk $RR_{temporal}$ and its confidence band for a total of 36 months from 2013 to 2015. The relative risk of tuberculosis has a seasonal periodicity. It is the most frequent period from February to May each year and most significant in March. It decreases from June to December. Figure 3 shows the temporal effects line for each year. Overall, the relative risk of tuberculosis decreases year by year.

#### Spatial–temporal distribution

**Figure 4:**
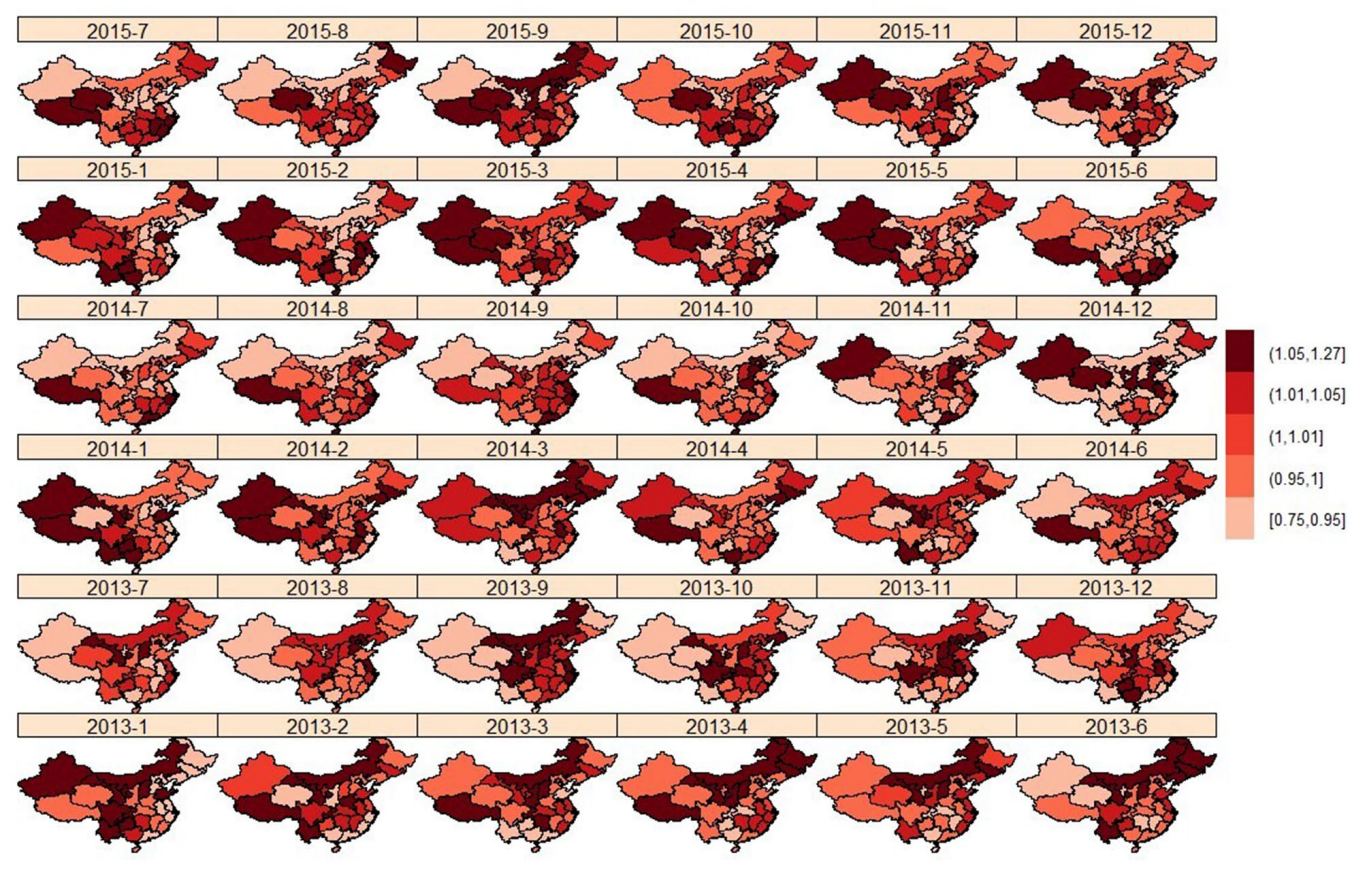
$$RR_{spatial-temporal}$$ distribution.

Interaction detection shows that there is a nonlinear enhancement between location and time. q(Location $\bigcap$ Time) = 1 is greater than the sum of q(Location) = 0.875 and q(Time) = 0.061. The interaction between spatial effect and temporal effect is nonlinearly enhancement. Spatial effect and temporal effect are not independent of each other. The spatial–temporal effect term $\delta$ represents a change that cannot be reflected by spatial and temporal effects alone. Figure 4 shows the relative risk in spatial–temporal effect $RR_{spatial-temporal}$ = $exp(\delta )$. From the figure, we can see the change of $RR_{spatial-temporal}$ in two adjacent regions over time. The temporal trend of the incidence risk in two adjacent regions is random. The temporal trend of the regions is also independent of the spatial structure. That is, the impact of unobserved variables on the relative risk of disease does not have the time $\times$ spatial structure, and can be separated into time effects and space effects. It can be seen in the figure that the spatial–temporal effect terms of Tibet and Qinghai increased more from 2013 to 2015, indicating that unobserved variables have a greater impact on Tibet and Qinghai. For example, the local medical conditions are not sufficiently developed.

## Discussion

This article investigates the influencing factors of the risk of tuberculosis and its spatial and temporal distribution. In general, the number and incidence of tuberculosis from 2013 to 2015 showed a downward trend as a whole. This rough result is satisfactory. The article gives a more rigorous analysis through four aspects. The results are expected to be professional for the research and control of tuberculosis in mainland China.

Spatially, the relative risk is different in different provinces. Compared with the existing studies^8,15,23^, Xinjiang, Guizhou, Guangxi and Hunan have been high-risk areas and hot spots. The result in this paper shows that the risk in Hainan is also high from 2013 to 2015. This may be because Hainan has a tropical marine climate with high humidity throughout the year, high rainfall, and long sunshine hours^24^. Therefore, it is necessary to strengthen the prevention and control of tuberculosis in Hainan Province. Early detection and early treatment of tuberculosis patients is necessary. Do a good job of disinfection and sterilization in public places and strengthen the popularization of tuberculosis prevention knowledge.

The relative risk of tuberculosis is different in time, season, and month. Studies have shown that the risk of Zhejiang Province is highest in April^18^, and then gradually decreases. The risk of morbidity in Yunnan is also highest in spring^25^. Overall, the relative risk of tuberculosis is higher in spring and lower in autumn and winter, so protective measures should be strengthened in spring. Remind the public to ventilate frequently and keep indoor air fresh. Strengthen physical exercise and improve immunity.

The existing study^23^ has shown that average temperature and average air pressure have negative effects on tuberculosis and average relative humidity has a positive effect, and the study^15^ has shown that average precipitation has a positive effect, which are consistent with the results from 2013 to 2015 studied in this article. The results of this paper show that precipitation has a positive effect on tuberculosis, which is consistent with the conclusions of existing studies^7,15^. This may be because tuberculosis is a chronic infectious disease caused by Mycobacterium tuberculosis^26^. Mycobacterium tuberculosis is more likely to survive in an environment with high humidity and precipitation, but not easy to survive in an environment with high temperature and pressure. The monthly average sunshine duration is particularly significant in promoting the risk of tuberculosis. The ultraviolet light contained in the light can harm human skin and eyes, and may cause a decline in human immunity and tuberculosis infection. The results of this study indicate that the duration of sunlight is an important factor affecting the risk of tuberculosis, so when studying the risk of tuberculosis, the duration of sunlight should be considered. The research in this paper shows that monthly GDP per capita has a positive effect on tuberculosis. This may be because the improvement of the economic level has made medical treatment more convenient, which is helpful for the diagnosis of tuberculosis. As GDP continues to increase, treatment levels and medical systems become more complete, the incidence of tuberculosis may decrease^8^.

Tibet is relatively remote, with large temperature differences between day and night and relatively long periods of sunlight. Although the results of this study show that Tibet is not in the five provinces with the high risk of tuberculosis from 2013 to 2015, more attention is still needed.

The meteorological factors selected in this paper are comprehensive, but there are still some shortcomings in this paper. First, this article only collected data for a total of 36 months from 2013 to 2015 and data for longer periods can be collected in future research. Second, this article only selects per capita GDP as a socio-economic factor, which can take into account hidden factors such as medical resources.

In summary, this article gives the influence of meteorological and economic factors on the relative risk of tuberculosis from 2013 to 2015 and analyzes the spatial and temporal distribution characteristics of the relative risk of tuberculosis. It is hoped that this will provide a certain theoretical basis for the prevention and control of tuberculosis.

## Methods

### Study area

The regions studied in this article are 31 provinces in mainland China, including Anhui, Beijing, Chongqing, Fujian, Gansu, Guangdong, Guangxi Zhuang Autonomous Region, Guizhou, Hainan, Hebei, Heilongjiang, Henan, Hubei, Hunan, Jiangsu, Jiangxi, Jilin, Liaoning, Inner Mongolia Autonomous Region, Ningxia Hui Autonomous Region, Qinghai, Shandong, Shanghai, Shanxi, Shaanxi, Sichuan, Tianjin, Xinjiang Uygur Autonomous Region , Tibet Autonomous Region, Yunnan and Zhejiang Province. In this article, the province is used as the research unit for the spatial–temporal analysis of tuberculosis.

### Data source

Tuberculosis surveillance data in 31 provinces from 2013 to 2015 came from the Chinese Center for Disease Control and Prevention, including the number and incidence of tuberculosis.

The meteorological data from 2013 to 2015 came from the China Meteorological Data Network, which included six variable meteorological data of 826 stations across the country for 36 months. The monthly meteorological data of 31 provinces from 2013 to 2015 were obtained by ordinary kriging interpolation method. Then the total monthly precipitation in the monthly meteorological data was converted into monthly average precipitation, and the total monthly sunshine duration was converted into the monthly average sunshine duration.

The quarterly GDP data for 2013–2015 came from the National Bureau of Statistics.First, the quarterly GDP was converted into monthly GDP, and then the monthly GDP of each province was converted into monthly per capita GDP.

### Model

Bernardinelli et al.^27^ proposed a Bayesian model to study spatial–temporal distribution of disease, also known as a Poisson log-linear model. This model studies the impact of spatial and temporal differences on the relative risk of a specific disease. That is the deviation from the overall relative risk in a region. The model include spatial effect and linear time effect terms, and the spatial effect and its corresponding time trend are random effects to reflect the overall relative risk level of a specific region. It also includes a separable space-time effect term, reflecting the temporal trends among regions. Knorr et al.^28^ changed the linear time effect term in the Poisson log-linear model to non-linear, including structured time effect and unstructured time effect, and changed the spatiotemporal effect interaction term to non-separable to adapt to more universal disease research. This spatial–temporal distribution model can better study and explain the spatial and temporal distribution characteristics of relative risk. In the studies^7,23^ of tuberculosis in mainland China, the time effect term is linear, and the spatiotemporal effect interaction term is not considered. The study of temporal and spatial–temporal distribution is not thorough enough.

The study of the spatial–temporal distribution of disease requires data from multiple regions, multiple times, and multiple influencing factors, and the amount of data is large. Compared with the MCMC method, the INLA algorithm proposed by Rue^29^ in 2009 has more powerful computing capabilities without losing the accuracy. Therefore, applying INLA algorithm to the study of the spatial–temporal distribution of diseases^30,31^ is an important method in epidemiology. In the paper, INLA algorithm was used to estimate the parameters of spatial–temporal distribution model.

Build the following spatial–temporal distribution model:

1 $$\begin{aligned} {\left\{ \begin{array}{ll} Y_{it}\sim Poisson(\lambda _{it})\\ \lambda _{it}=E_{it}\theta _{it}\\ log{(\theta _{it})}=b_0+u_i+v_i+{\sum }_k{\beta _kX_{kit}}+\gamma _t+\varphi _t+\delta _{it} \end{array}\right. } \end{aligned}$$

where $i=1,2, \ldots ,31$, $t=1,2, \ldots ,36$, $k=1,2, \ldots ,6$. $Y_{it}$ is the number of tuberculosis cases in the month *t*, following the Poisson distribution with the mean value of $\lambda _{it}$. $\lambda _{it}$ represents the average onset level on the area *i*. $E_{it}$ is the expected number of tuberculosis cases in the area *i* and month *t*, which is equal to the product of the number of people in area *i* and the national incidence rate in the month *t*, which represents the average national incidence. $\theta _{it}$ is the relative risk, which represents the risk of the area *i* compared to the overall risk of tuberculosis in the country. $b_0$ is the average log relative risk. $u_i$ is the spatial structured effect of the area *i*, which represents that the undefined features in the area *i* have a spatial structure and follow the conditional autoregressive distribution. $v_i$ is the spatial unstructured effect of the area *i*, which means that the undefined features in the area *i* do not have a spatial structure and follow a normal distribution. $u_i$ and $v_i$ can be regarded as hidden variables of area *i*^32^, which are related and unrelated to the location of the area, respectively. $X_{kit}$ is the value of the kth influencing factor in month *t* of area *i*. $\beta _k$ represents effect of the kth influencing factor. $\gamma _t$ is the structured effect of the month *t*, which means that the undefined features of the month *t* have a temporal structure and follow the second-order walking model. $\varphi _t$ is the unstructured effect of the month *t*, which means that the undefined features of the month *t* do not have a temporal structure and follow a normal distribution. $\gamma _t$ and $\varphi _t$ can be regarded as hidden variables of the month *t*, which are related to and irrelevant to the position of month *t*. $\delta _{it}$ is the spatial–temporal interaction effect in the area *i* and month *t*. $\delta$ follows the normal distribution and the precision matrix is $\kappa _{\delta }K_{\delta }$. $K_{\delta }$ is the structure matrix, $K_{\delta }=K_{v}\otimes K_{\varphi }$. The spatiotemporal interaction effect here represents that the unobserved variables in the area *i* and month *t* have no structure in the time $\times$ space. That is, the temporal incidence trend in two adjacent areas is random. The specific distribution of the above variables is as follows:

2 $$\begin{aligned} {\left\{ \begin{array}{ll} u_i|u_{-i}\sim Normal\left( \frac{1}{N_i}{\sum }_{j=1}^n{a_{ij}u_j},s^2_i\right) \\ v_i\sim Normal\left( 0,\frac{1}{\tau _{v}}\right) \\ \gamma _t|\gamma _{t-1},\gamma _{t-2}\sim Normal(2\gamma _{t-1}+\gamma _{t-2},\sigma ^2)\\ \varphi _t\sim Normal\left( 0,\frac{1}{\tau _{\varphi }}\right) \\ \delta _{it}\sim Normal\left( 0,\frac{1}{\tau _{\delta }}\right) , \end{array}\right. } \end{aligned}$$

where, $N_i=\#N(i)$, $s^2_i=\frac{1}{\tau _uN_i}$. Where $N_i$ is the number of neighbors in the area *i*. *N*(*i*) is the neighbors of the area *i*. If area *i* is adjacent to area *j*, $a_{ij}$ is equal to 1. Otherwise, $a_{ij}$ is 0. $a_{ii}$ is set to 0. $\tau _u$ is the precision parameter of the spatial structured effect and $\tau _v$ is the precision parameter of the spatial unstructured effect.

### Spatial stratified heterogeneity detector

China is huge and diverse in both environmental and socioeconomic determinants of TB prevalence. When analyzing the influence of factors on tuberculosis, it is necessary to detect spatial stratified heterogeneity. This article uses *q*-statistic^33^ to detect the spatial stratified heterogeneity of tuberculosis and the interaction of spatial and temporal effects. The *q*-statistic formula is as follows:

3 $$\begin{aligned} {q }=1-\frac{\sum _{h=1}^LN_h{\sigma _h^2}}{N\sigma ^2}, \end{aligned}$$

where, *h* is stratum and $h= 1, \ldots , L$. $N_h$ and *N* are the number of units in stratum *h* and the whole area, respectively; $\sigma _h^2$ and $\sigma ^2$ are the variances of the *Y* value of stratum *h* and the whole area, respectively. The value range of the *q*-statistic is [0,1]. The larger the value of *q*, the stronger the explanatory power of the factor to the dependent variable, otherwise the weaker. If *q* is equal to 0, it means that there is no relationship between the factor and the dependent variable. If *q* is 1, it means that the factor completely controls the spatial distribution of the dependent variable.

### Ethics declarations

This study does not involve human experiments, and uses public data from the China Centers for Disease Control and Prevention, so it was not approved by the Ethical Committee.

## Data Availability

Tuberculosis surveillance data generated during the current study are available in the Chinese Center for Disease Control and Prevention (http://www.phsciencedata.cn/Share/). The meteorological data are available in China Meteorological Data Network (http://data.cma.cn/). The quarterly GDP data are available in National Bureau of Statistics (http://www.stats.gov.cn/tjsj/). The software used in this paper is R: A Language and Environment for Statistical Computing, version 3.6.3 (https://www.R-project.org/).
